# A multimodal precision oncology platform integrating genomics, single-cell transcriptomics, and functional drug screening expands therapeutic options in pediatric brain tumors

**DOI:** 10.64898/2026.10.12.758619

**Published:** 2026-10-14

**Authors:** Xiaomeng Huang, Yi Qiao, Gage S. Black, Casey R. Sederman, Huiyi Yang, Teresa Musci, Alli Ludwick, Gongping He, Osama Youssef, Moises Gutierrez, Jingye Yang, Priya P. Chan, Trevor H. Memmott, Nicholas S. Whipple, Carol S. Bruggers, Aaron Quinlan, Philip J. Moos, Samuel H. Cheshier, Gabor T. Marth

## Abstract

Precision oncology for pediatric brain tumors mainly relies on clinically validated, targetable genomic alterations, which are often sparse, yield few matched therapies, and do not guide chemotherapy selection. We developed a tiered framework in which deep whole-genome sequencing and single-cell RNA sequencing extend targeted-therapy selection, with ex vivo functional drug screening supporting selection of both targeted therapies and chemotherapies. We applied it to 17 children with malignant CNS tumors. The approach expanded recommendations beyond genomics: functional screening provided every patient with at least one high-confidence targeted-therapy or chemotherapy nomination, while single-cell RNA sequencing identified an average of 37 druggable gene targets per patient, producing a fourfold increase in high-confidence targeted-therapy recommendations. In an exploratory analysis, functional classifications were directionally consistent with outcomes in 8 of 10 evaluable cases, although combination treatments and radiation precluded causal attribution. Single-cell profiling also identified subclone-specific vulnerabilities, supporting prospective evaluation of the platform’s feasibility and predictive utility.

